# Transcriptional profiles in bursal B-lymphoid DT40 cells infected with very virulent infectious bursal disease virus

**DOI:** 10.1186/s12985-016-0668-2

**Published:** 2017-01-13

**Authors:** Rong Quan, Shanshan Zhu, Li Wei, Jing Wang, Xu Yan, Zixuan Li, Jue Liu

**Affiliations:** Beijing Key Laboratory for Prevention and Control of Infectious Diseases in Livestock and Poultry, Institute of Animal Husbandry and Veterinary Medicine, Beijing Academy of Agriculture and Forestry Sciences, No. 9 Shuguang Garden Middle Road, Haidian District, Beijing, 100097 People’s Republic of China

**Keywords:** vvIBDV, Microarray, DT40 cells, Pathway analysis, Toll-like receptors, Inflammatory response, Bursa

## Abstract

**Background:**

Infectious bursal disease virus (IBDV) causes a highly contagious, immunosuppressive disease in chickens. The virus mainly infects immature B lymphocytes in the bursa of Fabricius (BF). Chicken B cell line DT40, an avian leukosis virus-induced B cell line, supports very virulent IBDV (vvIBDV) infection in vitro and thereby serves as a good model for investigating the infection and pathogenesis of this virus. However, a transcriptome-wide understanding of the interaction between vvIBDV and B cells has not yet been achieved. This study aimed to employ time-course DNA microarrays to investigate gene expression patterns in DT40 cells after infection with vvIBDV strain LX.

**Results:**

DT40 cells infected with vvIBDV exhibited alterations in the expression of many important host genes involved in signal transduction pathways, including MAPK signaling, PI3K/mTOR signaling, cell death and survival, BCR signaling, and antigen presentation. The changes in cellular mRNA levels identified by microarray analysis were confirmed for 8 selected genes using real-time reverse transcription-PCR. The upregulation of inflammatory cytokines and Toll-like receptors (TLRs) in the bursa of vvIBDV-infected chickens might involve excessive activation of the innate immune and inflammatory responses and contribute to tissue damage.

**Conclusions:**

The present study is the first to provide a comprehensive differential transcriptional profile of cultured DT40 cells in response to vvIBDV infection and further extends our understanding of the molecular mechanisms underlying vvIBDV infection and pathogenesis.

## Background

Infectious bursal disease virus (IBDV), a member of the family Birnaviridae, is a non-enveloped, double-stranded RNA virus composed of two segments: A (3.2 kb) and B (2.9 kb). Segment A encodes a precursor polyprotein that yields the mature VP2, VP4, and VP3 proteins as well as a non-structural protein, VP5; segment B encodes viral RNA-dependent polymerase protein VP1 [1]. Infection with IBDV results in infectious bursal disease, a highly contagious and immunosuppressive disease, in 3- to 15-week-old chickens and causes severe economic losses to the poultry industry worldwide. Two serotypes of IBDV have been recognized. Serotype I strains exhibit different degrees of pathogenicity and/or mortality in chickens, including attenuated, classical virulent, variant, and very virulent (vv) IBDV, whereas serotype II strains are non-pathogenic to chickens [2, 3]. The precursors of antibody-producing B lymphocytes in the bursa of Fabricius (BF) are the most important target cells for IBDV, and infection of the BF leads to B lymphocyte depletion and BF disruption [4]. IBDV-induced severe immunosuppression increases the susceptibility of IBDV-infected chickens to other infectious agents and reduces the immune response to vaccinations [5].

Increasing evidence indicates that IBDV infection differentially regulates host cellular genes and pathways correlated with virus replication and apoptosis. The IBDV VP2 protein may utilize host shock protein 90 in DF-1 cells and α4β1 integrin in BALB/c 3 T3 cells as components of a specific binding receptor that is essential for virus entry [6, 7]. The endosomal pathway and the Golgi complex are involved in IBDV replication [8], and IBDV infection induces apoptosis via the inducers VP2 and VP5 in vitro and in vivo [9, 10]. In SM and DF1 cells, the activation of the nuclear factor kappa B (NF-κB), c-Jun NH_2_-terminal kinase (JNK), p38 mitogen-activated protein kinase (MAPK) and phosphatidylinositol 3-kinase (PI3K)/Akt pathways by IBDV infection contributes to viral replication and virus-mediated apoptotic responses [11–13]. VP4 inhibits type I interferon via GILZ [14], and VP5 is involved in the release of viral particles [15]. However, more detailed information about the interactions between IBDV and host canonical pathways is needed to obtain an improved understanding of viral infection and pathogenesis.

Microarray is a high-throughput method for simultaneously assessing the mRNA transcriptional patterns of thousands of genes to evaluate virus-host cell interactions [16]. Wong et al. (2007) used a microarray technique to determine gene-expression profiles in chicken embryo fibroblast (CEF) cells after attenuated IBDV infection and observed a large degree of differential regulation of host cellular genes and pathways correlated with virus replication and apoptosis [17]. Some studies have also used RNA-Seq and comparative proteomic approach to explore mRNA and protein changes in the DF-1 and CEF cells with cell culture adapted IBDV infection [18, 19]. Only one proteomic approach was used to describe the differentially expresssion patterns of host cellular proteins in bursa of chickens by virulent IBDV infection [20]. However, CEF/DF1-adapted IBDV is attenuated, and virulent IBDV cannot grow on CEF cells; in particular, CEF cells are not target cells for IBDV infection. Therefore, the gene expression profiles of IBDV-infected CEF cells do not reflect authentic virulent IBDV infection under natural conditions. In addition to B cells, a variety of other immune cells are present in the bursa; thus, changes in protein expression might result from a mixture of various immune cells after IBDV infection. However, the avian leukosis virus-induced chicken B cell line DT40 [21] is susceptible to virulent IBDV infection, and thus, these cells can be used to explore the molecular pathogenesis of the virus [22, 23]. A comprehensive study of all host genes and virus-targeted host networks in B cells during IBDV infection, particularly vvIBDV infection, is lacking. Infection by vvIBDV strains results in high mortality in chickens, and vvIBDV is becoming the predominant clinical disease type in nearly all poultry-producing regions around the world [5]. However, a transcriptome-wide understanding of the interaction between vvIBDV and B cells has not yet been achieved.

In the present study, the vvIBDV strain LX was adapted to DT40 cells for growth and replication after serial passages. We then investigated the gene expression patterns of DT40 cells after infection by vvIBDV strain LX using Affymetrix microarrays. Functional and canonical pathway analyses of differentially expressed genes during vvIBDV infection validated many of the known as well as novel host genes corresponding to interesting functions and pathways. Infection of chickens with DT40-derived vvIBDV further confirmed the transcriptional profiles of inflammatory cytokines and TLRs after vvIBDV infection in vitro, which enhance the excessive activation of innate immune and inflammatory responses and contribute to tissue damage. These results will facilitate a better understanding of the interaction between vvIBDV and B cells and permit the further identification of key host factors for virus infection and pathogenesis.

## Results

### Confirmation of vvIBDV infection in DT40 cells

**Fig. 1:**
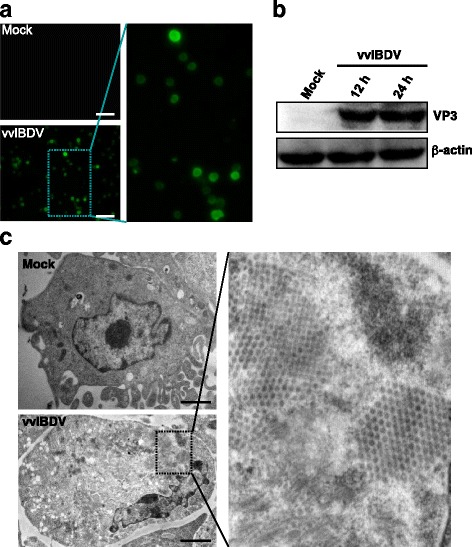
Determination of vvIBDV infection in DT40 cells. **a** Representative IFA staining of vvIBDV-infected cells exhibiting VP3 protein expression. DT40 cells infected for 24 h were fixed and analyzed by immunofluorescence staining with a guinea pig anti-VP3 polyclonal antibody. Partial enlargement is shown. Bars, 60 μm. **b** Whole-cell lysates prepared from cells infected with vvIBDV for 12 and 24 h were assayed by Western blotting for the presence of the VP3 protein. β-actin was used as a loading control. **c** Electron micrographs of vvIBDV-infected DT40 cells at 24 hpi. In the cytoplasm of the infected cells, approximately 60-nm viral particles were observed in crystalline arrays. Partial enlargement is shown. Bars, 2 000 nm

To determine whether vvIBDV strain LX is capable of replicating in DT40 cells, we passaged the strain in DT40 cells up to six generations to increase the viral titer. The DT40 cell-derived vvIBDV strain LX was titrated by inoculating 10-day-old specific pathogen-free (SPF) chick embryos, and the titer of the DT40-derived vvIBDV was 10^5.5^ 50% egg infectious dose (EID_50_)/0.2 ml. We compared the VP2 nucleotide sequence of this cell culture-derived vvIBDV with that of wild-type vvIBDV and found no changes after being passaged in chicken B-lymphoid DT40 cells (accession number: AF416624.1). An immunofluorescence assay (IFA) revealed green fluorescence in the vvIBDV strain LX-infected cells, indicating that the DT40 cells were successfully infected after vvIBDV inoculation (Fig. 1a). Total vvIBDV strain LX-infected cell lysates were subjected to 10% sodium dodecyl sulfate-polyacrylamide gel electrophoresis (SDS-PAGE) followed by western blotting to confirm the presence of the IBDV VP3 protein (Fig. 1b). DT40 cells after vvIBDV infection were also prepared for electron microscopy, which revealed the presence of viral particles with a diameter of 50–60 nm that exhibited a crystalline array in the cytoplasm of the infected cells (Fig. 1c). These results indicated that vvIBDV could infect and replicate successfully in DT40 cells with stable genetic characteristics. By contrast, neither IBDV viral protein expression nor IBDV particles were detected in mock-infected DT40 cells at any of the indicated time points (Fig. 1a-c and data not shown).

### Transcriptome analysis of DT40 cells in response to vvIBDV infection

**Fig. 2:**
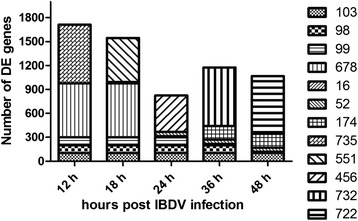
The number of differentially expressed genes at the indicated times after vvIBDV infection. The Y axis represents the number of genes. The X axis represents the time points after vvIBDV infection. There were 103 co-regulated DE genes at all time points

To assess the expression profile of mRNA transcripts in host cells, DT40 cells were inoculated with DT40-derived vvIBDV strain LX at a multiplicity of infection (MOI) of 5.0 EID_50_ for 12, 18, 24, 36, and 48 h. Total RNA was extracted from the infected cells at the indicated times post infection (pi) and hybridized with the Affymetrix GeneChip Chicken Genome Array. Cells incubated with phosphate-buffered saline (PBS) served as mock-infected controls. The microarray mRNA profiling was normalized to the internal endogenous control and cross-compared between vvIBDV-infected and mock-infected cells. Genes with a fold change (FC) > 2 or < 0.5 in expression were considered significantly differentially regulated. Among the 33,457 transcripts tested, we identified 4416 differentially regulated genes in the infected cells compared to the mock-infected control. Compared to the mock-infected controls, 1713, 1545, 824, 1175, and 1067 genes were significantly differentially expressed (DE) in vvIBDV-infected DT40 cells at 12, 18, 24, 36, and 48 h pi, respectively (Fig. 2). The fold changes in mRNA expression between the vvIBDV-infected and control groups ranged from 55.7 to 0.009. Furthermore, approximately 70% of the total regulated genes exhibited steady downregulation after vvIBDV infection: 1235 of 1713, 1106 of 1545, 526 of 824, 941 of 1175, and 666 of 1067 genes at 12, 18, 24, 36, and 48 h, respectively. This finding indicated that vvIBDV infection compromised host biological activities via a dominating global downregulation. For each individual gene, variable temporal expression distributions were also observed at different time points after vvIBDV infection, which might reflect host responses to vvIBDV infection that depend on viral gene expression and/or interaction with specific host material or machinery at different stages of vvIBDV infection.

### Validation of mRNA expression by qRT-PCR

**Fig. 3:**
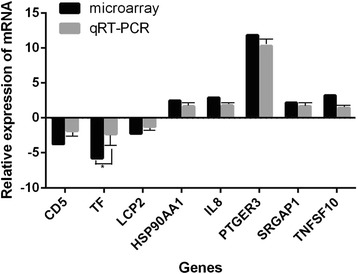
qRT-PCR confirmation of 8 randomly selected mRNAs from the microarray analysis. Average fold changes of gene expression in DT40 cells at 24 h following vvIBDV infection, as determined by qRT-PCR and microarray analysis, are shown. The expression of selected mRNAs in vvIBDV-infected cells together with the expression of mock-infected controls was validated by qRT-PCR using a pair of specific primers and a probe for each mRNA. The fold change was calculated based on endogenous control normalization. Data are expressed as the mean ± SD of triplicate reactions for each gene transcript

To validate the differentially regulated mRNAs identified by hybridization microarray, eight genes with different expression levels were selected for qRT-PCR analysis. These genes are involved in different functional groups or pathways, including immune system process (CD5, LCP2, and IL8), signal transduction (PTGER3, HSP90AA1, and SRGAP1), apoptosis (TNFSF10), and transport (TF). All selected genes were successfully amplified from total cellular RNA of mock- and vvIBDV-infected DT40 cells (at 24 h), and the expression patterns corresponded to those determined by microarray. Of the five upregulated mRNAs evaluated (see Table 1), all displayed upregulation by independent qRT-PCR assay. Similarly, the three downregulated mRNAs exhibited downregulation in the tested samples. As illustrated in Fig. 3, identical differential expression patterns were observed by qRT-PCR and microarray, further confirming the reliability of the microarray analysis.

**Table 1:** Significant pathways of differentially expressed genes in IBDV-infected DT40 cells

12 h^a^		18 h		24 h		36 h		48 h	
Name	No.	Name	No.	Name	No.	Name	No.	Name	No.
Metabolic pathways	107	Metabolic pathways	107	Metabolic pathways	48	Metabolic pathways	67	Metabolic pathways	60
MAPK signaling pathway	27	MAPK signaling pathway	32	Endocytosis	16	MAPK signaling pathway	26	MAPK signaling pathway	21
Regulation of actin cytoskeleton	24	Regulation of actin cytoskeleton	22	Regulation of actin cytoskeleton	15	Endocytosis	21	Focal adhesion	17
Neuroactive ligand- receptor interaction	19	Focal adhesion	21	Focal adhesion	14	Regulation of actin cytoskeleton	19	Endocytosis	17
Focal adhesion	19	Endocytosis	20	Tight junction	13	Focal adhesion	18	Tight junction	15
Endocytosis	19	Cytokine-cytokine receptor interaction	18	Neuroactive ligand- receptor interaction	13	Cytokine-cytokine receptor interaction	18	Regulation of actin cytoskeleton	15
Cytokine- cytokine receptor interaction	18	Neuroactive ligand-receptor interaction	17	MAPK signaling pathway	12	Tight junction	15	Ubiquitin mediated proteolysis	14
Tight junction	17	Purine metabolism	16	Vascular smooth muscle contraction	11	Neuroactive ligand- receptor interaction	14	Neuroactive ligand- receptor interaction	13
VEGF signaling pathway	14	Vascular smooth muscle contraction	15	Calcium signaling pathway	10	Calcium signaling pathway	13	Calcium signaling pathway	13
RNA degradation	14	Lysosome	15	Ubiquitin mediated proteolysis	9	Vascular smooth muscle contraction	12	Vascular smooth muscle contraction	11
Cell adhesion molecules (CAMs)	14	Tight junction	14	Lysosome	9	Jak-STAT signaling pathway	12	Cell adhesion molecules (CAMs)	11

^a^hours post LX vvIBDV infection

### Functional gene clusters and canonical pathway analysis

**Fig. 4:**
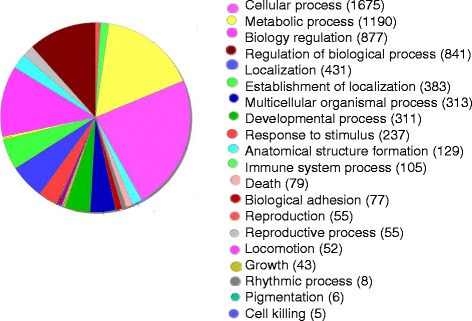
Categories of annotated DE genes based on biological process GO terms. Gene ontology classification of differentially regulated genes detected during vvIBDV infection. The genes were primarily clustered into 22 functional groups with variable numbers. Many categories shared the same genes

Gene ontology analysis of the microarray data was performed to identify the main host functions in response to vvIBDV infection. The differentially altered host genes fell into diverse functional categories and were classified into a range of biological processes using GO terms. As shown in Fig. 4, all genes with fold change (FC) > 2 or < 0.5 were clustered into 22 functional groups. The main biological processes at all time points after vvIBDV infection included cellular process, metabolic process, biological regulation, regulation of biological process, and localization. Many categories shared the same genes. The KEGG pathways associated with DE genes after vvIBDV infection are listed in Table 1. The canonical pathways included metabolic pathways, cytokine/chemokine receptor interaction, MAPK signaling pathway, regulation of actin cytoskeleton, endocytosis, and neuroactive ligand receptor interaction. These pathways may play roles in host antiviral protective responses and IBDV pathogenesis.

### Altered expression of genes involved in cytokine pathways and the inflammatory response

The microarray data indicated that TLR expression in chicken B cells exhibited a distinct pattern in response to vvIBDV infection, with decreases in the expression of TLR2, TLR6, and TLR7. By contrast, TLR15 expression was upregulated 8.4- and 6.1-fold at 24 and 36 h pi, respectively. Differential changes in the expression of CCL19, CCL20, IL-8, IL-15, and Trail/TNF (ligand) superfamily members TNFSF10 and TNFSF15 in DT40 cells were also observed at different time points after vvIBDV infection. Several interferon-inducible genes involved in anti-viral immune responses, including the ubiquitin-like protein modifiers ISG12-1 and ISG20L2, tripartite motif proteins TRIM8, TRIM23, TRIM24, and TRIM29, and ubiquitin-specific peptidases USP22, USP32, and USP49, were also differentially regulated in response to vvIBDV infection. These genes with known or predicted functions are all involved in cytokine pathways and the inflammatory response. These results suggest that vvIBDV infection greatly modulates the immune function of DT40 cells.

### Cytokine and TLR profiles in BF of chickens inoculated with DT40-derived vvIBDV

**Fig. 5:**
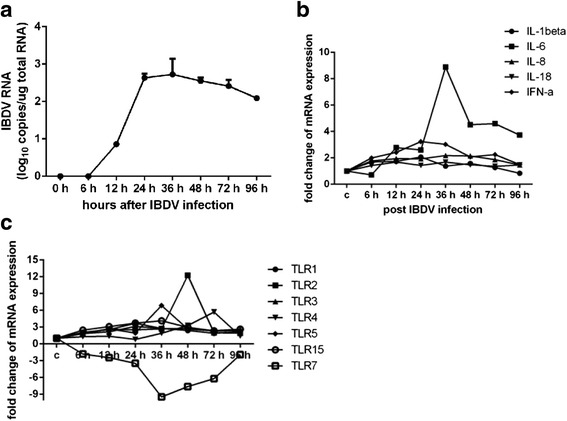
Cytokine and TLR profiles in the bursa of SPF chickens inoculated with DT40 cell-derived vvIBDV. **a** Quantification of viral loads in the bursa of vvIBDV-infected chickens by real-time RT-PCR. Total RNA isolated from the IBDV-infected bursa at the indicated time points was analyzed by absolute real-time RT-PCR analysis of VP2 RNA. The values are the means of the results for the three chickens in each group; error bars show the standard deviations. Expression levels of inflammatory cytokines **b** and TLRs (**c**) in the bursa of vvIBDV-infected chickens are shown. Total RNA isolated from bursa samples of infected or sham-infected chickens (*n* = 3 per group) at all time points after vvIBDV infection were analyzed by real-time RT-PCR analysis to detect inflammatory cytokine and TLR transcripts. The data were normalized to the amount of GAPDH mRNA. The data are expressed as percentages of the normalized value for the vvIBDV-infected bursa versus the sham-infected bursa; the values are the means ± SD of values from three chickens. The statistical analysis was performed by comparing data from sham-infected chickens. *P < 0.05

IBDV predominately infects B lymphocytes in the BF of chickens. As noted above, IBDV induced different profiles of immune genes in DT40 B cells in vitro. We hypothesized that mRNA expression data from DT40 cells in vitro might enable the prediction of the in vivo pathogenesis of vvIBDV infection. We therefore used DT40-derived vvIBDV to inoculate SPF chickens to evaluate the expression of selected host immune and inflammatory genes in vivo, including the cytokine and TLR profiles from the microarray results. SPF chickens were inoculated with 10^5^ EID_50_ of DT40-derived vvIBDV and observed daily. A cohort exhibiting the typical clinical signs of disease, such as anorexia, depression, ruffled feathers, prostration, or diarrhea, was observed during the 4-day experiment. At the indicated time points post-inoculation, bursa samples from the sham- and vvIBDV-infected chickens were collected to analyze cytokine and TLR profiles. To confirm viral replication, we used qRT-PCR to detect viral loads in homogenized bursal samples of the vvIBDV-infected chickens using a pair of primers specific for the VP2 gene (Table 2). The virus loads increased rapidly during the first two days pi and persisted at high levels until the end of the experiment (96 h; Fig. 5a). By contrast, no IBDV nucleotide signals were detected in the bursa of sham-inoculated chickens at any of the experimental time points (data not shown). Histological evaluation of the bursas from the chickens infected with DT40-derived vvIBDV revealed severe necrosis and disintegration of lymphocytes in follicles, and a large number of heterophils, macrophages and reticular cells infiltration from 48 to 96 h pi (data not shown).

**Table 2:** Gene primers for qRT-PCR

Gene_Symbol	Gene	Sequence
TF-F	Transferrin	AACAACCTCAGGGACCTCAC
TF-R		GTCCAAGCTAATGGCATCTG
LCP2-F	Lymphocyte cytosolic protein 2	TGAATCACCAACGGAAGAAA
LCP2-R		ACTGGAGGCTGATGTGATGA
TNFSF10-F	Tumor necrosis factor (ligand) superfamily, member 10	TGGCCGTCACCTACATCTAC
TNFSF10-R		TCAGCCACTCTGTCTTTGCT
CD5-F	CD5 molecule	ACAGGAGGCTGATGAAGAGG
CD5-R		TGAGCGTAATCGTTGTCTCC
IL8-F	Interleukin 8	GGAAGAGAGGTGTGCTTGGA
IL8-R		TAACATGAGGCACCGATGTG
HSP90AA1-F	Heat shock protein 90 kDa alpha (cytosolic), class A member 1	CCTGATTCCAAACAAGCACG
HSP90AA1-R		TTCTCCGCAACAAGGTAAGC
PTGER3-F	Prostaglandin E receptor 3 (subtype EP3)	GGATCATGTGCGTCCTGTC
PTGER3 -R		CACGGCTGTCAAGAAGAAAT
SRGAP1 F	SLIT-ROBO Rho GTPase activating protein 1	CAGATTGGGAGGTCAGGAGA
SRGAP1 R		GAAAACGGAAGCATTGGTTG
GAPDH-F	Glyceraldehyde-3-phosphate dehydrogenase	GAGGGTAGTGAAGGCTGCTG
GAPDH-R		CATCAAAGGTGGAGGAATGG
IFN-αF	Interferon α	GACATGGCTCCCACACTACC
IFN-αR		AGGCGCTGTAATCGTTGTCT
IL-1βF	Interleukin 1β	GGATTCTGAGCACACCACAGT
IL-1βR		TCTGGTTGATGTCGAAGATGTC
IL-6 F	Interleukin 6	ATCCGGCAGATGGTGATAAA
IL-6R		CCCTCACGGTCTTCTCCATA
IL-18 F	Interleukin 18	ACGTGGCAGCTTTTGAAGAT
IL-18R		GCGGTGGTTTTGTAACAGTG
TLR-1 F	Toll-like receptor 1	GCTGTGTCAGCATGAGAGGA
TLR-1R		GTG GTACCTCGCAGGGATAA
TLR-2 F	Toll-like receptor 2	GAA AGTTCCCCCTTTTCCAG
TLR-2R		AGAGTGCAGAAGGTCCCTGA
TLR-3 F	Toll-like receptor 3	CCTCCTTGGGACACCTGA AA
TLR-3R		ATTCCGCAGTGGATGAAAAG
TLR-4 F	Toll-like receptor 4	GCTGGGCAAAGTGAAAAGAG
TLR-4R		TAAGAACAGCCCGTTCATCC
TLR-5 F	Toll-like receptor 5	CCACTGCTGGAGGATTTGTT
TLR-5R		TCCAGGATGGAATCTCCA AG
TLR-7 F	Toll-like receptor 7	AGAGACTGGCTTCCAGGACA
TLR-7R		CAGCTGAACATACCGGGACT
TLR-15 F	Toll-like receptor 15	CCATCAACAGCCTGGAAACT
TLR-15R		CCTGGTTTCTGACCAAGGAA
LXVP2F	VP2	AGAGCTGTGGCCGCAGACAAT
LXVP2R		TGGATAGTTGCCACCGTGGAT

The transcriptional profile results for selected innate immune response genes in the bursa of DT40 cell-derived vvIBDV-infected chickens are presented in Fig. 5b and c. Significantly higher levels of inflammatory cytokines IFN-α, IL-6, and IL-8 were observed in the vvIBDV-inoculated group (*n* = 3 per group) compared to the sham-infected group at the indicated time points. Of these genes, IL-6 was the most highly expressed (upregulation of 11.9-fold at 36 hpi). The levels of IL-1β and IL-18 expression increased slightly in vvIBDV-infected chickens, whereas there was no significant difference in the expression levels of IL-10 and IL-17 between the two groups (data not shown).

TLR2, TLR4, TLR5, and TLR7 were differentially expressed to a greater extent than TLR1, TLR3, and TLR15 at 36, 48, and 72 hpi. Compared to the sham-inoculated chickens, the fold changes in TLR5 and TLR7 expression in the bursa of vvIBDV-infected chickens were 6.9 and −9.5, whereas TLR1, TLR2, TLR3, and TLR4 expression increased by 2.7-, 2.7-, 2.8-, and 1.9-fold, respectively, at 36 h pi. The fold change in TLR2 expression in the bursa of vvIBDV-infected chickens was 12.2 at 48 h pi, whereas TLR4 expression increased by 5.7-fold at 72 h pi. Of these genes, only TLR7 was downregulated.

Taken together, the cytokine and TLR expression profiles in the bursa of the DT40 cell-derived vvIBDV-infected chickens indicated that the innate immune and inflammatory responses were induced by vvIBDV infection, further confirming the observations for vvIBDV infection of DT40 cells in vitro.

## Discussion

In the present study, we used Affymetrix microarrays to obtain an overview of the transcriptional changes induced by vvIBDV infection in a chicken bursa-derived B cell line, DT40. The comprehensive transcriptomic profile of the vvIBDV pathogenic process in target host cells revealed that several major aspects of chicken B cell function were affected by vvIBDV infection, including cell survival and apoptosis, mitogen-activated protein kinase (MAPK) signaling, BCR signaling, MHC class II molecules, and B-cell differentiation. The transcriptional profiles of inflammatory cytokines and TLRs after vvIBDV infection in vitro were consistent with the gross and histopathological signs, including the inflammatory responses in DT40 cell-derived vvIBDV-infected chickens.

### MAPK signal transduction pathways

Specific signal transduction pathways stimulated by many stimuli, including lipopolysaccharide (LPS), bacteria, and virus infection, play roles in regulating cell proliferation and differentiation, cytokine production, apoptosis, cell survival and immune responses [24, 25]. IBDV exploits the p38 MAPK signal transduction machinery to induce the production of inducible nitric oxide synthase (iNOS), IL-8 and cyclooxygenase-2 (COX-2), thereby contributing to macrophage activation and bursa inflammatory responses [11]. JNK, one of the major components of MAPK signal transduction pathways, is required for IBDV replication and the regulation of virus-induced apoptosis in DF-1 cells [10]. As observed for IBDV infection in macrophages [10] and DF-1 cells [13], the present study also demonstrated that neutrophil chemotactic factor IL-8 increased in DT40 cells after vvIBDV infection; furthermore, the mRNA level of transcription factor ATF2 involved in the JNK signaling pathway persistently increased from 12 h pi. These results demonstrate that vvIBDV infection can induce the activation of JNK and p38 MAPK and their downstream targets in DT40 cells, contributing to virus replication and inflammatory responses.

### Autophagy and apoptosis pathways

Autophagy is a key physio-pathological process that has a complex relationship with cell death and survival. In the present study, the expression of PI3K3R1 and mTOR was suppressed. By contrast, MAPK1 was upregulated in response to vvIBDV infection, which might indicate inhibition of the Akt/mTOR pathway. Inhibition of the PI3K/Akt/mTOR pathway can induce pro-autophagic activity. In agreement with a recent report regarding IBDV-infected DF1 cells [26], there may be a link between the activation of autophagy and the MTOR signaling pathway in vvIBDV-infected DT40 cells. An essential autophagy protein, Beclin-1 [27–30], exhibited a 2.25-fold increase at 12 h pi and remained upregulated to similar levels at 18 h pi; upregulation of Bcl-2, an anti-apoptotic protein that can inhibit the activation of pro-apoptotic proteins, was also observed at both the these time points. Following vvIBDV infection, Fas and Apaf-1 exhibited upregulation of 2.3- and 2.1-fold, respectively, at 48 h pi; these factors are involved in the FAS-FASL interaction and the p53 signaling pathway to contribute to the apoptotic response. In addition to these genes, apoptosis-induced cysteine-aspartic acid peptidase (caspase) 3 exhibited mild 1.78-fold upregulation. These expression pattern data may indicate the induction of autophagy and inhibition of apoptosis in the early stages of vvIBDV infection. It will be novel and interesting to focus on the unexpected relationship of autophagy and apoptosis in vvIBDV-infected B cells in future studies. These results will help us to understand the discrepancies observed among different IBDV infection experimental setups or cell sources, as reported previously for Vero, CEF, and DT40 cells [15, 31, 32].

### Regulation of B-cell responses

The present study revealed that the biological processes of BCR signaling, endocytosis, vesicle transport, antigen capture and MHC class II expression were influenced by vvIBDV infection in DT40 cells. CD72, which has been reported to regulate BCR-mediated signals both positively and negatively, was downregulated at 12 and 18 h pi. DT40 B cell antigen receptor (BCR) requires the recruitment of cytosolic protein tyrosine kinases (PTK) to trigger signaling pathways [33, 34]. The JAK-STAT and PTK Lyn-STAT pathways are two independent cascades involved in BCR signaling. In our study, both PTK2 and JAK1 were decreased at 12, 18, and 24 h after vvIBDV infection. Raftlin is a B cell-specific raft protein that plays a role in B-cell activation [35], and Lyn kinase belongs to the specific raft proteins. RFTN2 was significantly downregulated at 18 and 24 h pi. Disruption of the raftlin gene may reduce the content of specific raft proteins and cell proliferation and attenuate BCR signaling. The defect in the JAK-STAT and PTK Lyn-STAT pathways may underlie the suppression of aberrant STAT activation in immune disorders. Asl2 exhibited suppression of −2.17 and −2.08 FC at 12 and 18 h pi, respectively, whereas Rab22a displayed upregulation at 48 h pi. The products of Asl2 act as regulators that stimulate early endosome fusion, and Rab22a, which localizes to the plasma membrane and early endosomes, is a small GTPase that plays key roles in vesicle transport. Atp6v1c1 is one of the components of the vesicle-type proton pump and plays a role in the acidification of endocytic vesicles in preparation for their fusion with lysosomes to facilitate antigen hydrolysis. Several genes involved in MHC class II expression were also influenced by vvIBDV infection. For example, Sec61a1 exhibited a significant 22-fold upregulation at 12 h pi; the Sec61 translocation complex is involved in the translocation of nascent MHC class II peptide chains into the lumen of the endoplasmic reticulum. Calmegin (Clgn), a Ca^++^-binding chaperone protein that regulates the assembly of MHC class II molecules, exhibited −11 and −25-fold downregulation at 18 and 24 h pi, respectively. The expression of CD8a, CD74, and BCL6, which are regulators of MHC class II molecules and B-cell differentiation [36], was downregulated during early vvIBDV infection. DT40 cells are immunological cells that highly express TLR7 and can initiate an antiviral immune response [37, 38]. IL18 induces IFN-γ and MHC class II Ag [39], and TLR-7 and IL18 expression levels were suppressed in vvIBDV-infected DT40 cells. Our observations suggest an important role for abnormal BCR-induced signaling events and B cell antigen processing and presentation in host-vvIBDV interactions.

In addition, the downregulation of TUBD1 and microfilament-associated protein CNN3 associated with the cytoskeleton was detected in DT40 cells following vvIBDV infection, as also observed in CEF cells [17]. Although the transcription factor NFIA was inhibited, ATF2 was activated by vvIBDV infection. The discrepancies in mRNA signatures in the present and previous reports may be due to differences in the cell types and virus strains used.

### Toll-like receptors

TLRs are essential sensors of microbial infection and play a pivotal role in the activation of innate immunity by recognizing specific patterns of a variety of microbial components. TLR15, an avian-specific TLR, may recognize invading microbes, including viruses and bacteria [40]. TLR3 and TLR7, which are the only TLRs implicated in antiviral responses in chickens, are involved in the recognition of some RNA viruses, such as avian influenza virus and IBDV infection [41–43]. In the present study, differential TLR gene expression was observed in vvIBDV-infected DT40 cells. vvIBDV infection of DT40 cells led to the downregulation of TLR7 and upregulation of TLR15; these results were further confirmed by TLR expression profiles in the bursa of DT40 cell-derived vvIBDV-inoculated chickens. Strikingly, TLR2 and TLR4 were downregulated in vvIBDV-infected DT40 cells but upregulated in vvIBDV-inoculated bursa. This may be related to the presence of other immune cells, such as macrophages and infiltrating heterophils and T cells, in the vvIBDV-inoculated bursa. Furthermore, other TLRs, such as TLR1, TLR3, and TLR5, were upregulated in the vvIBDV-inoculated bursa, and the upregulation of these receptors may enhance the sensitivity of B cells to other pathogens, thereby augmenting the host immune response. However, over-stimulation of the innate immune response might lead to tissue damage. Therefore, TLR activation may contribute to the pathogenesis of vvIBDV infection in chickens, but the role of TLR-mediated innate immune activation in the pathogenesis of vvIBDV infection remains to be elucidated.

### Inflammatory response

The recruitment of inflammatory cells into pathological tissues is modulated by a variety of cytokines and chemokines. The vvIBDV-infected DT40 cells in our study exhibited differential regulation of the mRNA expression of CCL19, CCL20, IL-8, IL-15, IL-18, and the Trail/TNF (ligand) superfamily members TNFSF10 and TNFSF15. The present study also revealed that the inflammatory gene profile of the vvIBDV-inoculated bursa differs slightly from that of vvIBDV-infected DT40 cells. Research has suggested that IBDV infection can lead to the activation of macrophages [44]. In chickens, activated macrophages produce a variety of pro-inflammatory cytokines [44, 45], and modulation of the inflammatory reaction via the upregulation of a few inflammatory cytokines (IL-1β, IL-6, IL-8, and IL-18) might contribute to bursa lesions after vvIBDV infection. IL-1β serves as a potent stimulator of heterophil recruitment and is essential for pathogen infection; IL-8 is a chemoattractant for heterophils and monocytes produced following acute infection [46]. Excessive activation of the inflammatory response can contribute to organ damage in combination with the cytokine storm and exaggerate disease induced by viral or bacterial infection. Thus, it is reasonable to speculate that the development of bursa damage in chickens suffering from vvIBDV infection may result from the recruitment of inflammatory cells and the activation of macrophages.

## Conclusions

In conclusion, this is the first study to use DNA microarrays to measure the expression levels of host genes in DT40 B cells with vvIBDV infection. The results presented here provide evidence that differentially expressed mRNAs in DT40 cells after vvIBDV infection are involved in a variety of biological regulation processes, including signal transduction pathways related to the immune response. In vivo experiments in chickens further confirmed the observed inflammatory and innate responses induced by vvIBDV in DT40 cells in vitro, and IL-1β, IL-18, TLR1, TLR2, and TLR3 are the predominant DE genes after vvIBDV infection. Taken together, the present study establishes a comprehensive differential transcription profile of vvIBDV infection in chicken bursal B-lymphoid DT40 cells and enhances our further understanding of the molecular mechanism underlying vvIBDV infection and pathogenesis.

## Methods

### Virus and cells

The DT40 cell line, an avian leukosis virus-induced chicken B cell line, was maintained in RPMI 1640 medium supplemented with 8% heat-inactivated fetal bovine serum (FBS), 2% heat-inactivated chicken serum, 10% tryptose phosphate broth, 1 mM sodium pyruvate, 100 U/ml penicillin G, and 100 μl/ml streptomycin at 37 °C in a humidified 5% CO_2_ incubator. IBDV strain LX [47], a vvIBDV strain, was propagated inSPF chickens to maintain the pathogenicity of the virus. vvIBDV strain LX was inoculated and passaged in DT40 cells as described elsewhere [23]; virus that had been passaged six times in DT40 cells was used to study mRNA expression patterns after infection. The virus titer was determined by the (EID_50_. For infection, DT40 cells seeded the day before were infected with vvIBDV strain LX at an MOIof 5. Mock-infected DT40 cells were used as negative controls.

### Indirect fluorescence assay (IFA)

DT40 cells grown in 96-well plates were infected with vvIBDV strain LX at an MOI of 5 EID_50_. At 24 h, the cells were washed with PBS and fixed with 4% paraformaldehyde (PFA). After three washes, the cells were incubated at 37 °C for 1 h with guinea pig anti-VP3 polyclonal antibody diluted in 3% bovine serum albumin (BSA)-PBS. After three further washes, the cells were incubated at 37 °C for 1 h with goat anti-guinea pig fluorescein isothiocyanate (FITC)-conjugated antibody (Sigma) diluted in 3% BSA-PBS. The cells were washed three times with PBS and examined by fluorescence microscopy.

### Western blot analysis

Whole-cell lysates from DT40 cells after vvIBDV infection at the indicated time points were prepared using a Nuclear Extract Kit (Active Motif) according to the manufacturer’s protocol. The whole-cell lysates were diluted in 2 × sample buffer and boiled for 5 min. Twenty micrograms of each extract was resolved by 10% SDS-PAGE and blotted onto nitrocellulose (NC) membranes using a semidry transfer cell (Trans-Blot SD; Bio-Rad). The membranes were blocked for 2 h at room temperature (RT) in TBST blocking buffer (20 mM Tris–HCl [pH 7.4], 150 mM NaCl, 0.1% Tween 20) containing 5% skim milk powder to prevent nonspecific binding and then incubated with guinea pig anti-VP3 antibody at RT for 2 h. The membranes were then washed three times with TBST buffer and incubated for 1 h at RT with horseradish peroxidase (HRP)-conjugated anti-guinea pig antibody (Sigma) diluted in blocking buffer. Immunoreactive bands were visualized by enhanced chemiluminescence (Kodak Image Station 4000R). A monoclonal antibody against β-actin (Santa Cruz Biotechnology) was used as a loading control for Western blotting.

### Electron microscopy

DT40 cells (2–3 × 10^6^) were collected by scraping at the indicated time points after vvIBDV infection and centrifuged at 1,000 × g for 10 min at 4 °C. The pellets were washed in PBS and fixed in 2.5% glutaraldehyde. The cells were then post-fixed in 1% OsO_4_ and embedded in EPON-812. Ultrathin sections were cut and examined under a Hitachi H-700 electron microscope.

### mRNA extraction and microarray assay

The Affymetrix GeneChip Chicken Genome Array, which can evaluate the gene expression of 33,457 chicken and viral transcripts, was used in this analysis. DT40 cells were infected with vvIBDV strain LX at an MOI of 5 EID_50_ and collected at the indicated time points post-infection (pi). Total RNA was isolated using an miRNeasy Mini Kit (QIAGEN). Both the integrity and concentration of RNA were evaluated using an Agilent Bioanalyzer 2100 (Agilent Technologies), and any RNA samples that exhibited degradation were excluded from the present study. After quality assessment, the RNA was used for cDNA synthesis. The cDNA was then transcribed to cRNA, which was tagged with biotin using the GeneChip 3′IVT Express Kit (Affymetrix). Array hybridization and washing were performed using the GeneChip Hybridization, Wash and Stain Kit in a Hybridization Oven 645 and a Fluidics Station 450 according to the manufacturer’s instructions. Slides were scanned using a GeneChip Scanner 3000 and Command Console Software 3.1 with default settings. The raw data were normalized using the MAS 5.0 algorithm, Gene Spring Software 11.0 (Agilent technologies). mRNA hybridization and data capture were performed by Shanghai Bio Corporation (Shanghai, China). Hierarchical clustering, gene ontology, and pathway analysis were performed using SAS (Shanghai Bio Analysis System).

### Microarray data accession number

Array data obtained in this study are deposited in the Gene Expression Omnibus database (GEO, http://www.ncbi.nlm.nih.gov/geo/). GEO accession number is GSE71888.

### Validation of differentially regulated mRNAs in the microarray by real-time reverse transcription-polymerase chain reaction (RT-PCR)

To detect selected mRNAs, total cell RNA was prepared from vvIBDV-infected DT40 cells at the indicated time points for real-time RT-PCR using an iScript one-step RT-PCR kit with SYBR Green (Bio-Rad) according to the manufacturer’s instructions. Primers for the selected genes were synthesized by Sangon Biotech, and the sequences are presented in Table 2. The RT-PCR parameters consisted of 50 °C for 10 min and 95 °C for 5 min, followed by 40 cycles of 95 °C for 10 s and 55 °C for 30 s; each sample was analyzed in triplicate. The relative amount of selected host mRNA was normalized to that of GAPDH mRNA in the same sample. The data were analyzed by the 2-∆∆CT method. The results are shown as fold changes.

### Infection of chickens with DT40-derived vvIBDV

A total of 30 25-day-old SPF leghorn chickens were purchased from Vital River Laboratories, Beijing, China. The chickens were randomly allocated to two groups and housed in isolation rooms under controlled temperature (21–23 °C), humidity (30–50%), and lighting (12 h light/12 h dark) and fed sterilized food and water *ad libitum*. One group (21 of chickens) was inoculated ocularly and intranasally with DT40-derived vvIBDV strain LX at 10^5^ EID_50_ in a total volume of 500 μl, whereas the other group (9 of chickens) served as a negative control and was sham inoculated with 500 μl of DT40 cell supernatant. Clinical signs of the chickens were monitored daily during the 4-day experiment. At 0, 6, 12, 24, 36, 48, 72, and 96 h pi, the chickens were humanely euthanized via cervical dislocation following isoflurane anesthesia, and BF samples were collected from both groups to assay the expression levels of IL-1β, IL-6, IL-8, IL-10, IL-17, IL-18, TLR1, TLR2, TLR3, TLR4, TLR5, TLR7, and TLR15 using real-time RT-PCR. The primers are listed in Table 2; GAPDH was used as an endogenous control. Absolute real-time RT-PCR was performed to determine IBDV virus loads in bursa samples collected at the indicated time points after infection. Additionally, bursa samples were collected and fixed by immersion in 2.5% glutaraldehyde-polyoxymethylene solution. The fixed tissues were dehydrated, embedded in paraffin wax, sectioned at 4-μm thickness, and then stained with hematoxylin and eosin (HE) for histopathological examination.

### Statistical analysis

Statistical analyses were performed using two-tailed Students’ *t*-tests (paired). *P* < 0.05 was considered statistically significant.
